# Efficient Silencing of Gene Expression by an ASON–Bulge–DNAzyme Complex

**DOI:** 10.1371/journal.pone.0018629

**Published:** 2011-04-07

**Authors:** Jianzhong Yi, Chengqian Liu

**Affiliations:** Institute of Animal Husbandry Veterinary Sciences, Shanghai Academy of Agricultural Sciences, Shanghai, China; City of Hope National Medical Center and Beckman Research Institute, United States of America

## Abstract

**Background:**

DNAzymes are DNA molecules that can directly cleave cognate mRNA, and have been developed to silence gene expression for research and clinical purposes. The advantage of DNAzymes over ribozymes is that they are inexpensive to produce and exhibit good stability. The “10-23 DNA enzyme” is composed of a catalytic domain of 15 deoxynucleotides, flanked by two substrate-recognition domains of approximately eight nucleotides in each direction, which provides the complementary sequence required for specific binding to RNA substrates. However, these eight nucleotides might not afford sufficient binding energy to hold the RNA substrate along with the DNAzyme, which would interfere with the efficiency of the DNAzyme or cause side effects, such as the cleavage of non-cognate mRNAs.

**Methodology:**

In this study, we inserted a nonpairing bulge at the 5′ end of the “10–23 DNA enzyme” to enhance its efficiency and specificity. Different sizes of bulges were inserted at different positions in the 5′ end of the DNAzyme. The non-matching bulge will avoid strong binding between the DNAzyme and target mRNA, which may interfere with the efficiency of the DNAzyme.

**Conclusions:**

Our novel DNAzyme constructs could efficiently silence the expression of target genes, proving a powerful tool for gene silencing. The results showed that the six oligo bulge was the most effective when the six oligo bulge was 12–15 bp away from the core catalytic domain.

## Introduction

At least four different approaches have been used for gene silencing to date, these include: gene knockout by homologous recombination [1]; antisense inhibition by the use of synthetic oligonucleotides that hybridize with DNA or RNA [2], [3], [4]; cleavage of target mRNA by ribozymes or DNAzymes [5]; and RNA interference by inducing the endogenous siRNA process pathway [6], [7]. Studies by Paterson *et al.* (1977) demonstrated that gene expression could be modified through the use of exogenous nucleic acids that were complementary to target RNAs. Zamecnik and Stephenson (1978) inhibited the replication of Rous sarcoma virus by the use of a short antisense DNA oligonucleotide (ASON) that was reverse complementary to a particular transcript of the virus [8]. ASONs or ASON analogs are often employed for *in vivo* antisense applications due to their stability and nuclease resistance [9], [10], [11]. Generally, these molecules block gene expression by hybridizing to the target mRNA, resulting in subsequent double-helix formation which inhibits transcript processing or translation. Furthermore, cellular RNases are able to bind to the DNA–RNA duplex and hydrolyze the mRNA, resulting in increased mRNA transcript degradation [12], [13], [14].

Breaker and Joyce (1994) made the assumption that because RNA and DNA are similar chemical compounds, DNA molecules with enzymatic activity could also be developed as ribozymes. This proposition led to the development of a DNA enzyme identified from a library of >1,000 different DNA molecules by successive rounds of *in vitro* selective amplification. The selected molecule was designated the “10–23 DNA enzyme,” and was composed of a catalytic domain of 15 deoxynucleotides flanked by two substrate-recognition domains of ∼8 nucleotides on each side [15], [16], [17]. The recognition sequences provided the specificity for binding to target RNA substrates and supplied the binding energy required to hold the RNA substrate with the DNAzyme [18], [19]. Although DNAzymes are less costly and more stable than ribozymes, they still encounter problems such as stability, efficiency and specificity to target mRNAs [20], [21]. Their complexes with RNA are less stable than the corresponding RNA:RNA duplexes, hence, mismatches might be generated during their interactions with non-target mRNAs, such as the mRNAs from other members of a gene family, resulting in non-specific cleavage [22], [23], [24], [25]. In this study, we introduced new bulge structure at the 5′ end of the “10–23” deoxyribozyme to enhance the efficiency and specificity of the DNAzyme.

## Results

### Design and synthesis of ASON–Bulge–DNAzymes

**Figure 1:**
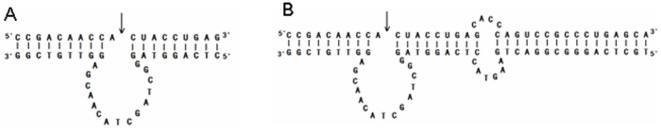
Schematic of the structure of ASON-Bulge-DNAzyme. **A**. Regular DNAzyme. **B**. ASON-Bulge-DNAzyme. A 15 base AS-ON was inserted at the 5′ end of “10-23”deoxyribozyme to target the mRNA substrate and an unmatched loop between the DNAzyme sequence and ASON sequence was designed.

In this study, we designed different sizes of bulges at different positions at the 5′ end of the regular DNAzyme. A non-matching bulge will avoid strong binding between the DNAzyme and target mRNA, which may interfere with the efficiency of the DNAzyme. Furthermore, the bulge structure would activate cellular RNases to hydrolyze the mRNA, resulting in increased mRNA transcript degradation. We introduced different sized oligo loops between the DNAzyme core sequence and the ASON sequence (Figure 1).

### ASON–Bulge–DNAzyme significantly reduced the expression of eGFP

**Figure 2:**
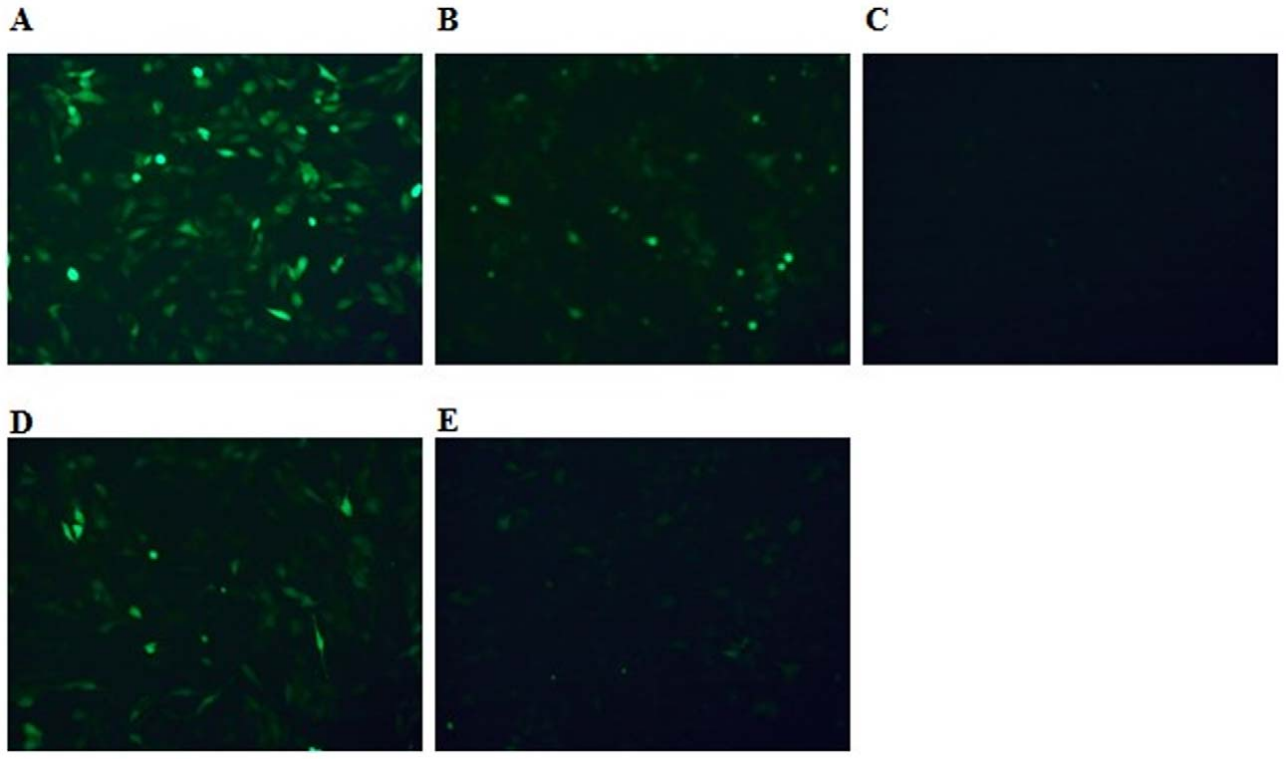
Transfection of ASON-Bulge-DNAzyme resulted in significantly reduced eGFP expression. BHK21 cells were co-transfected with 0.5 µg pECFP-C1 plasmid and 0.25 µg of DNAzymes **A**. pEGFP control; **B**. Regular DNAzyme-1; **C**. ASON-Bulge-DNAzyme-1; **D**. Regular DNAzyme-2; **E**.ASON-Bulge-DNAzyme-2. Calibration bar for **A-E**, 100 µm.

To assess the efficiency of the ASON–Bulge–DNAzyme complex, the ASON–Bulge–DNAzymes were chemically synthesized and co-transfected with pEGFP-C1 plasmid into BHK21 cells. As shown in Figure 2 (A, B and C), the ASON–Bulge–DNAzymes exhibited a significant reduction in enhanced green fluorescent protein (eGFP) expression compared with the regular DNAzyme.

To examine whether the efficiency of the ASON–Bulge–DNAzyme was site limited, we synthesized another ASON–Bulge–DNAzyme targeting a different site, the eGFP mRNA. As shown in Figure 2 (D and E), the new structure also exhibited much higher efficiency than the regular DNAzyme. These results demonstrated that the bulge-containing DNAzyme was more effective in gene silencing compared with the regular DNAzyme.

### The effect of size and position of the bulge on the efficiency of gene silencing

**Figure 3:**
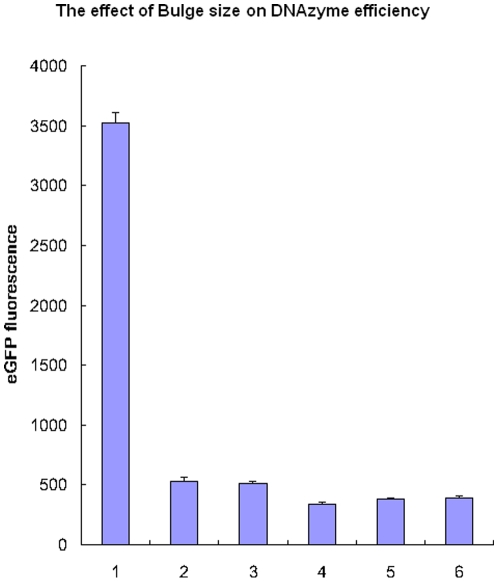
The effect of different bulge size on DNAzyme efficiency. Cells were cotransfected with 0.5 µg pECFP-C1 plasmid and 0.25 µg of DNAzymes. **1**. pEGFP control; **2**. Regular DNAzyme; **3**. 3nt-Bulge-DNAzyme; **4**. 6nt-Bulge-DNAzyme; **5**. 9nt-Bulge-DNAzyme; **6**. 12nt-Bulge-DNAzyme. The error bars denote Standard deviation (SD).

**Figure 4:**
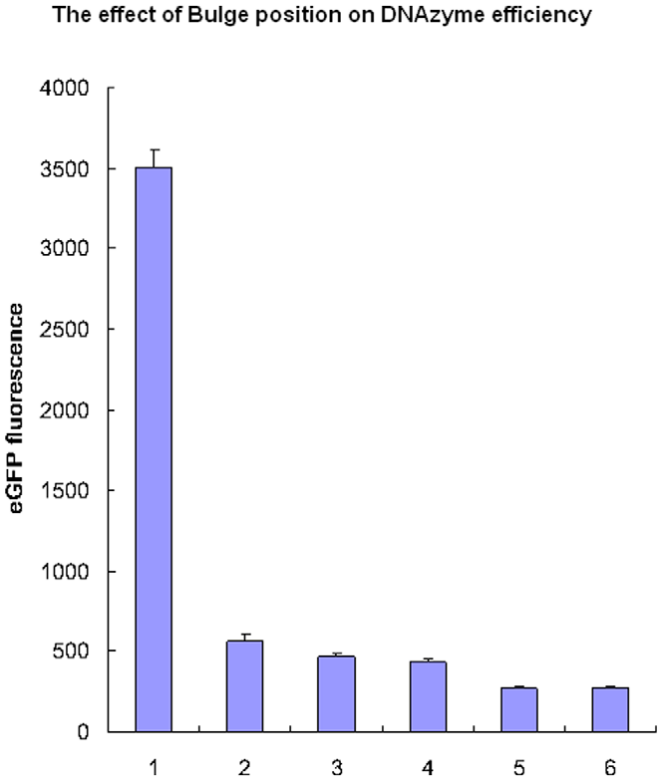
The effect of different bulge positions on DNAzyme efficiency. Cells were co-transfected with 0.5 µg pECFP-C1 plasmid and 0.25 µg of DNAzymes. **1**. pEGFP control; **2**. Regular DNAzyme; **3**. 6nt; **4**. 9nt; **5**. 12nte; **6**.15 nt. The error bars denote Standard deviation (SD).

To assess the inhibition effect of different bulge constructs, we tested eGFP expression levels by a GFP fluorescence assay. DNAzymes with three, six, nine and 12 oligo bulges were designed and co-transfected with pEGFP-C1 plasmid into BHK21 cells. The results showed that the six oligo bulge was the most effective (Figure 3). Furthermore, the DNAzyme was most effective when the six oligo bulge was located 12–15 bp away from the core catalytic domain (Figure 4).

### ASON–Bulge–DNAzyme inhibited the expression of eGFP over a long time period

**Figure 5:**
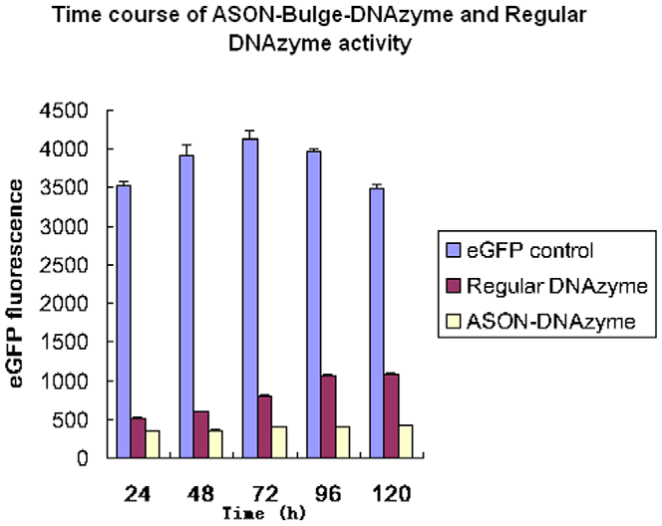
Quantitative assessment of the silencing efficiency of ASON-Bulge-DNAzyme by GFP fluorescence assay. BHK21 cells were harvested and lysed in 200 µl lysis buffer (0.1 M Tris-HCl, 0.1% Triton X-100, 2 mM EDTA, pH 7.8). Thefluorescence of eGFP was measured using a luminometer. The error bars denote Standard deviation (SD).

To quantitatively assess the inhibition effect of these constructs, we tested eGFP expression levels at different time points by a GFP fluorescence assay. The results further confirmed that the ASON–Bulge structure could not only enhance the efficiency of the DNAzyme, but could also sustain the inhibitory effect for longer compared with the regular DNAzyme (Figure 5).

### ASON–Bulge–DNAzyme decreased eGFP expression via the mRNA degradation pathway

**Figure 6:**
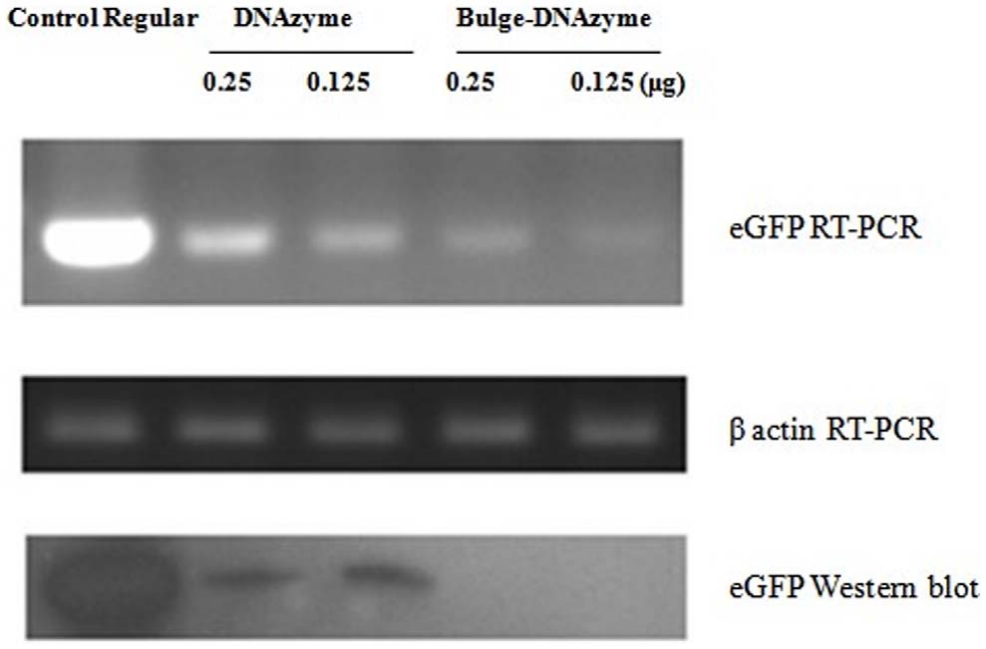
Semi-quantitative RT-PCR and Western blot analysis of the mechanism of ASONBulge-DNAzyme. **A**. RT-PCR of eGFP. **B**. RT-PCR of β-actin. **C**. Western blot of eGFP.

To test whether the ASON–Bulge–DNAzyme decreased eGFP expression by virtue of the mRNA degradation pathway, a semi-quantitative RT-PCR assay was employed to test the mRNA level of eGFP. The results confirmed that the ASON–Bulge structure could enhance the efficiency of the DNAzyme by decreasing the eGFP mRNA level. Western blot analysis further confirmed the efficiency of the ASON–Bulge–DNAzyme (Figure 6).

### ASON–Bulge–DNAzyme significantly reduced the expression of luciferase

**Figure 7:**
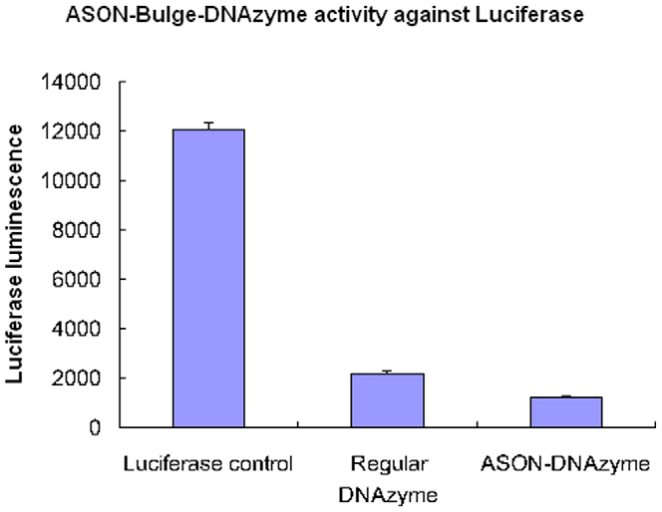
Quantitative assessment of the silencing efficiency of ASON-Bulge-DNAzyme against luciferase. BHK21 cells were harvested and lysed by in 200 µl lysis buffer (0.1 M Tris-HCl, 0.1% Triton X-100, 2 mM EDTA, pH 7.8). The luciferase activity was measured using a luminometer. The error bars denote Standard deviation (SD).

To assess whether the ASON–Bulge–DNAzyme was gene specific, we synthesized an ASON–Bulge–DNAzyme against firefly luciferase. The results showed that this structure was more effective than the regular DNAzyme (Figure 7).

### ASON–DNAzyme was effective against, and specific to, endogenous genes

**Figure 8:**
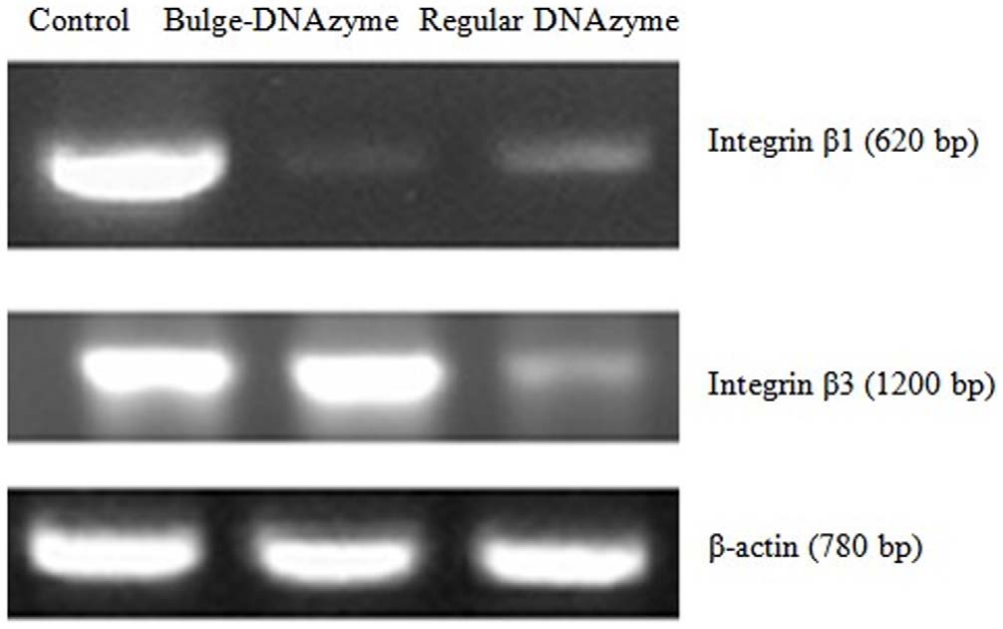
ASON-Bulge-DNAzyme can efficiently silence the expression of endogenous cellular genes. BHK21 cells were transfected with 0.25 µg of DNAzyme against integrin β1 in each well of a 24-well plate. RT-PCR was performed at 36 h after transfection. ASON-DNAzyme significantly reduced the mRNA level of integrin β1, but not integrin β3.

To test whether the ASON–Bulge–DNAzyme was effective against endogenous genes, we selected integrins as target molecules. Integrins are a large family of heterodimeric transmembrane glycoproteins that attach cells to extracellular matrix proteins of the basement membrane or to ligands on other cells. There are many types of integrins on the cell surface relating to different functions. We designed an ASON–Bulge–DNAzyme against the endogenous integrin β1 (targeted sequence β1: 5′-GATTCTCCAGAAGGTGGTTTCGATGCCATCATGCAAGTTGCAGTTTGTGGATCACTGATT-3′, β3: 5′-GATGCCCCAGAGGGTGGCTTTGATGCCATCATGCAGGCTACAGTCTGTGATGAAAAGATT-3′). The chemically synthesized DNAzyme was used to transfect HepG2 cells. As shown in Figure 8, the mRNA level of integrin β1 was significantly decreased, while the mRNA level of integrin β3 was unchanged compared with the regular DNAzyme. These results showed that this structure not only enhanced the efficiency of the DNAzyme, but also increased its specificity by minimizing the side effects, i.e., digestion of non-target mRNA.

## Discussion

Although RNA interference is the most widely used tools for gene silencing, The instablity, short half-life, inducing undesired IFN responds and other drawbacks *per se* limits its application in biology studies, clinical trials, and antivirus treatments [26], [27], [28], [29]. The advantage of DNAzymes over ribozymes is that they are inexpensive to produce and inherently more stable [30], [31]. DNAzymes can be designed to cleave virtually any RNA target by selecting appropriate flanking sequences [32], [33]. The recognition domains provide specificity and binding energy, however, if the binding strength is too strong or too weak between the DNAzyme and the substrate this can be antagonistic to catalysis [34], [35], [36].

DNAzymes face the same challenges of ribozymes such as the ability to be targeted to the cell of interest, intracellular localization, the ability to hybridize with the mRNA target, and the ability to achieve a sufficient intracellular concentration while avoiding interacting with non-target mRNAs [37], [38]. Within living cells, mRNA transcripts exist in secondary structures and interact with cytoplasmic proteins, therefore, some of the mRNA sequences are hidden and only partial sequences are accessible [39]. mRNAs exist in several cellular compartments, including the cytoplasm, nucleus and nucleolus. To suppress gene expression, DNAzymes must be colocalized to the same intracellular compartment as their target mRNA [40], [41]. Antisense RNA inserted within a variable region of ribosomal RNA (rRNA) has been shown to enhance ribozyme efficiency due to colocalization of rRNA with mRNA [42], [43].

In this study, we introduced complementary oligonucleotides (ASONs) at the 5′ end of DNAzymes to enhance the specificity of targeting to the mRNA substrate. To avoid too high a binding strength between the DNAzyme and the target mRNA, we introduced a nonpairing bulge structure into the DNAzyme. The formation of a bulge structure ensured that the target mRNA was easily hydrolyzed. Our results demonstrated that the incorporation of an ASON and a mispairing bulge in the DNAzyme conferred higher efficiency and specificity in gene silencing.

## Materials and Methods

### Design and synthesis of the ASON–Bulge–DNAzyme

A 15 base ASON was inserted at the 5′ end of the “10-23” deoxyribozyme. To avoid too high a binding strength between the DNAzyme and the target mRNA which might interfere with DNAzyme efficiency, we introduced a nonpairing loop at the 5′ end of the DNAzyme. The oligonucleotides used for this were as follows:

Regular DNAzyme-1 against eGFP:

5′-CTCAGGTAGGGCTAGCTACAACGAGGTTGTCGG-3′

ASON-Bulge-DNAzyme-1 against eGFP:

5′-TGCTCAGGGCGGACTGAAGTACCTCAGGTAGGGCTAGCTACAACGAGGTTGTCGG-3′

### Size of the bulge in the annealing arm complex of the DNAzyme

Bulges of differing sizes were incorporated into the complex using the following oligonucleotides:

The 3 nt Bulge–DNAzyme:

5′-TGCTCAGGGCGGACTGTACCTCAGGTAGGGCTAGCTACAACGAGGTTGTCGG-3′

The 6 nt Bulge–DNAzyme:

5′-TGCTCAGGGCGGACTGAAGTACCTCAGGTAGGGCTAGCTACAACGAGGTTGTCGG-3′

The 9 nt Bulge–DNAzyme:

5′-TGCTCAGGGCGGACTGAAGCGATACCTCAGGTAGGGCTAGCTACAACGAGGTTGTCGG-3′

The 12 nt Bulge–DNAzyme:

5′-TGCTCAGGGCGGACTGAAGTGACGTTAACCTCAGGTAGGGCTAGCTACAACGAGGTTGTCGG-3′

### Position of the bulge in the annealing arm of the DNAzyme

The position of the bulge in the complex was altered using the following oligonucleotides:

1. Bulge–6 nt–DNAzyme:

5′-TGCTCAGGGCGGACTGAAGTACCTCAAGGGCTAGCTACAACGAGGTTGTCGG-3′

2. Bulge–9 nt–DNAzyme:

5′-TGCTCAGGGCGGACTGAAGTACCTCAGGTAGGGCTAGCTACAACGAGGTTGTCGG-3′

3. Bulge–12 nt–DNAzyme:

5′-CTTGCTCAGGGCGGAAGTACGGTGCTCAGGTAGGGCTAGCTACAACGAGGTTGTCGG-3′

4. Bulge–15 nt–DNAzyme:

5′-TCTTTGCTCAGGGATCTACCTGGGTGCTCAGGTAGGGCTAGCTACAACGAGGTTGTCGG-3′

DNAzymes against Luciferase were constructed using the following oligonucleotides:

Regular DNAzyme against Luciferase:

5′-AGCATCGAGAGGCTAGCTACAACGACCGTTGT-3′

ASON–Bulge–DNAzyme against Luciferase:

5′-GGGCAATTTACCACGAGCAATAGCATCGAGAGGCTAGCTACAACGACCGTTGT-3′.

DNAzymes against integrin β1 were constructed using the following oligonucleotides:

Regular DNAzyme against integrin β1:

5′-TTGCATGAGGCTAGCTACAACGAGGCATCG-3′

ASON–Bulge–DNAzyme against integrin β1:

5′-GTGATCCACAAACTGCAAGTACAACTTGCATGAGGCTAGCTACAACGAGGCATCG-3′

### Semi-quantitative RT-PCR analysis of eGFP

Two pairs of specific primers were designed according to the sequence of the eGFP gene and the β-actin gene. The primers were synthesized by Shanghai Generay Biotech Co., Ltd (China).

eGFP-F1: 5′-CTGGACGGCGACGTAAACGG-3′

eGFP-R1: 5′-CACGAACTCCAGCAGGACCAT-3′

β-actin-F1: 5′-GGCTCCGGCATGTGCAAGGC-3′

β-actin-R1: 5′-GAAGGTAGTTTCGTGGATGCC-3′

cDNA was synthesized using 4 µl of the total RNA, 4 µl of 5×reverse transcriptase buffer, 1 µl of 10 mM dNTPs, 1 µl of eGFP-R1 primer (10 µM), 1 µl of β-actin-R1 primer (10 µM), 1 µl of RNase inhibitor (20 U/µl), 1 µl of MMLV reverse transcriptase (5 U/µl) and 7 µl of RNase-free H_2_O in a total volume of 20 µl for 1 h at 42°C. The PCR conditions were as follows: 1 cycle at 94°C for 5 min; followed by 30 cycles of 30 s at 94°C, 30 s at 55°C and 60 s at 72°C. A final elongation step of 10 min at 72°C was included.

### Semi-quantitative RT-PCR analysis of integrin genes

Two pairs of specific primers were designed according to the sequence of the integrin β1 gene and the integrin β3 gene. The primers were synthesized by Shanghai Generay Biotech Co., Ltd.

Integrin β1-F1: 5′-ATCCCAGAGGCTCCAAAGATAT-3′

Integrin β1-R1: 5′-CACCAAGTTTCCCATCTCCAG-3′

Integrin β3-F1: 5′-AGGATTACCCTGTGGACATC-3′

Integrin β3-R1: 5′-CCGTGATCTTGCCAAAGTCAC-3′

cDNA was synthesized using 4 µl of the total RNA, 4 µl of 5×reverse transcriptase buffer, 1 µl of 10 mM dNTPs, 1 µl of integrin β1-R1 primer (10 µM), 1 µl of integrin β3-R1 primer (10 µM), 1 µl of RNase inhibitor (20 U/µl), 1 µl of MMLV reverse transcriptase (5 U/µl) and 6 µl of RNase-free H_2_O in a total volume of 20 µl for 1 h at 42°C. The PCR conditions were as follows: 1 cycle at 94°C for 5 min; followed by 35 cycles of 40 s at 94°C, 30 s at 55°C and 60 s at 72°C. A final elongation step of 10 min at 72°C was included.

### Cell culture and transfection

BHK-21 cells (from ATCC) were cultured in Dulbecco's modified Eagle's medium (DMEM; GIBCO, USA) containing 10% fetal bovine serum (FBS) supplemented with 1% penicillin–streptomycin (GIBCO). Cells were cultured in 24-well plates and transfected by Lipofectamine 2000 (Invitrogen, USA). The ratio of DNAzyme to Lipofectamine was 1 µg:2 µl. Cells were co-transfected with 0.5 µg of pECFP-C1 plasmid and 0.25 µg of siRNA expression cassettes in each well.

### Western blot analysis

BHK-21 cells were harvested 36 h after eGFP DNAzyme transfection. They were then washed with PBS three times, and lysed with 100 µl of lysis buffer. Total cellular protein was determined using the BioRad Protein Assay reagent (Bio-Rad Laboratories). The eGFP was detected using mouse anti-GFP antibody (Abmart, China). The blot was developed using ECL Plus (Amersham Biosciences).

### Reporter gene assay

BHK-21 cells were harvested and lysed with 200 µl of lysis buffer (0.1 M Tris, 0.1% Triton X-100, 2 mM EDTA, pH 7.8). The eGFP fluorescence was analyzed using a luminometer (Modulus, Turner Biosystems, USA).

## References

[1] Bronson SK, Smithies O (1994). Altering mice by homologous recombination using embryonic stem cells.. J Biol Chem.

[2] Paterson BM, Roberts BE, Kuff EL (1977). Structural gene identification and making by DNA:mRNA hybrid-arrested cell-free translation.. Proc Natl Acad Sci USA.

[3] Mizuno T, Chou MY, Inouye M (1984). A unique mechanism regulating gene expression: translational inhibition by a complementary RNA transcript (micRNA).. Proc Natl Acad Sci USA.

[4] Izant JG, Weintraub H (1984). Inhibition of thymidine kinase gene expression by anti-sense RNA: a molecular approach to genetic analysis.. Cell.

[5] Pieken WA, Olsen DB, Benseler F, Aurup H, Eckstein F (1991). Kinetic characterization of ribonuclease-resistant 2′-modified hammerhead ribozymes.. Science.

[6] Fire A, Xu S, Montgomery MK, Kostas SA, Driver SE (1998). Potent and specific genetic interference by double-stranded RNA in *Caenorhabditis elegans*.. Nature.

[7] Kennerdell JR, Carthew RW (1998). Use of dsRNA-mediated genetic interference to demonstrate that frizzled and frizzled 2 act in the wingless pathway.. Cell.

[8] Zamecnik PC, Stephenson ML (1978). Inhibition of Rous sarcoma virus replication and cell transformation by a specific oligodeoxynucleotide.. Proc Nat Acad Sci USA.

[9] Agrawal S, Zhao Q (1998). Antisense therapeutics.. Curr Opin Chem Biol.

[10] Galderisi U, Cascino A, Giordano A (1990). Antisense oligonucleotides as therapeutic agents.. J Cell Physiol.

[11] Gewirtz AM (1998). Antisense oligonucleotide therapeutics for human leukemia.. Curr Opin Hematol.

[12] Guvakova MA, Yakubov LA, Vlodavsky I, Tonkinson JL, Stein CA (1995). Phosphorothioate oligodeoxynucleotides bind to basic fibroblast growth factor, inhibit its binding to cell surface receptors, and remove it from low affinity binding sites on extracellular matrix.. J Biol Chem.

[13] Gao WY, Han FS, Storm C (1992). Phosphorothioate oligonucleotides are inhibitors of human DNA polymerases and RNase H: implications for antisense technology.. Mol Pharmacol.

[14] Egholm M, Buchardt O, Christensen L, Behrens C, Freier SM (1993). PNA hybridizes to complementary oligonucleotides obeying the Watson-Crick hydrogen-bonding rules.. Nature.

[15] Breaker RR, Joyce GF (1994). A DNA enzyme that cleaves RNA.. Chem Biol.

[16] Santoro SW, Joyce GF (1997). A general purpose RNA cleaving DNAzyme.. Proc Natl Acad Sci USA.

[17] Breaker RR (2000). Making catalytic DNAs.. Science.

[18] Sun L-Q, Cairns MJ, Saravolac EG (2000). Catalytic nucleic acids: from lab to applications.. Pharmacol Rev.

[19] Silverman SK (2010). DNA as a versatile chemical component for catalysis, encoding, and stereocontrol.. Angew Chem Int Ed Engl.

[20] Santoro SW, Joyce GF (1998). Mechanism and utility of an RNA-cleaving DNA enzyme.. Biochemistry.

[21] Kurreck J, Birgit B, Jahnel R, Erdmann VA (2002). Comparative study of DNA enzyme and ribozymes against the same full-length messenger RNA of the vanilloid receptor subtype I. J Biol. Chem.

[22] Cairns MJ, Hopkins TM, Witherington C, Wang L, Sun L-Q (1999). Target site selection for an RNA-cleaving catalytic DNA.. Nat Biotechnol.

[23] Emilsson GM, Breaker RR (2002). Deoxyribozymes New activities and new applications.. Cellular and Molecular Life Sciences.

[24] Nielsen PE, Egholm M, Berg RH, Burchardt O (1993). Peptide nucleic acids (PNAs): potential antisense and anti-gene agents.. Anticancer Drug Res.

[25] Ota N, Warashina M, Hirano K, Hatanaka K, Taira K (1998). Effects of helical structures formed by the binding arms of DNAzymes and their substrates on catalytic activity.. Nucleic Acids Res.

[26] Castanotto D, Rossi JJ (2009). The promises and pitfalls of RNA-interference-based therapeutics.. Nature.

[27] Lares MR, Rossi JJ, Ouellet DL (2010). RNAi and small interfering RNAs in human disease therapeutic applications.. Trends Biotechnol.

[28] Pecot CV, Calin GA, Coleman RL, Lopez-Berestein G, Sood AK (2011). RNA interference in the clinic: challenges and future directions.. Nat Rev Cancer.

[29] Perrimon N, Ni JQ, Perkins L (2010). In vivo RNAi: today and tomorrow.. Cold Spring Harb Perspect Biol.

[30] Liu Q, Paroo Z (2010). Biochemical principles of small RNA pathways.. Annu Rev Biochem.

[31] Breaker RR (2004). Natural and engineered nucleic acids as tools to explore biology.. Nature.

[32] Chan SH, Stoddard BL, Xu SY (2011). Natural and engineered nicking endonucleases—from cleavage mechanism to engineering of strand-specificity.. Nucleic Acids Res.

[33] Franzen S (2010). Expanding the catalytic repertoire of ribozymes and deoxyribozymes beyond RNA substrates.. Curr Opin Mol Ther.

[34] Dass CR, Saravolac EG, Li Y, Sun L-Q (2002). Cellular uptake, distribution and stability of 10-23 deoxyribozymes.. Antisense Nucleic Acid Drug Dev.

[35] Schubert S, Gul DC, Grunert HP, Zeichhardt H, Erdmann VA (2003). RNA cleaving ‘‘10-23’’ DNAzymes with enhanced stability and activity.. Nucleic Acids Res.

[36] Werner M, Uhlenbeck OC (1995). The effect of base mismatches in the substrate recognition helices of hammerhead ribozymes on binding and catalysis.. Nucleic Acids Res.

[37] Lam JC, Withers JB, Li Y (2010). A complex RNA-cleaving DNAzyme that can efficiently cleave a pyrimidine-pyrimidine junction.. Journal of molecular biology.

[38] Wong JP, Christopher ME, Salazar AM, Sun LQ, Viswanathan S (2010). Broad-spectrum and virus-specific nucleic acid-based antivirals against influenza.. Front Biosci (Schol Ed).

[39] Schlosser K, Li Y (2009). DNAzyme-mediated catalysis with only guanosine and cytidine nucleotides.. Nucleic Acids Res.

[40] Mastroyiannopoulos NP, Uney JB, Phylactou LA (2010). The application of ribozymes and DNAzymes in muscle and brain.. Molecules.

[41] Bennett CF, Chiang MY, Chan H, Shoemaker JE, Mirabelli CK (1992). Cationic lipids enhance cellular uptake and activity of phosphorothioate antisense oligonucleotides.. Mol Pharmacol.

[42] Schubert S, Kurreck J (2004). Ribozyme- and deoxyribozyme-strategies for medical applications.. Curr Drug Targets.

[43] Tong-Hong Wang, Wan-Ting Li, Szu-Hsien Yu, Lo-Chun Au (2008). The Use of 10-23 DNAzyme to Selectively Destroy the Allele of mRNA with a Unique Purine-Pyrimidine Dinucleotide.. Oligonucleotides.

